# Supplementary material for: The interactome of intact mitochondria by cross-linking mass spectrometry provides evidence for coexisting respiratory supercomplexes
Source: Mol Cell Proteomics. 2017 Dec 8;17(2):216–32. doi: 10.1074/mcp.RA117.000470 (PMC5795388; doi:10.1074/mcp.RA117.000470)
Supplement: Supplemental Data [file supp_RA117.000470_133922_0_supp_23978_fzffwf.zip › spectra_annotation/mito_spectra_annotation/210-1-17-1-13-1.pdf]

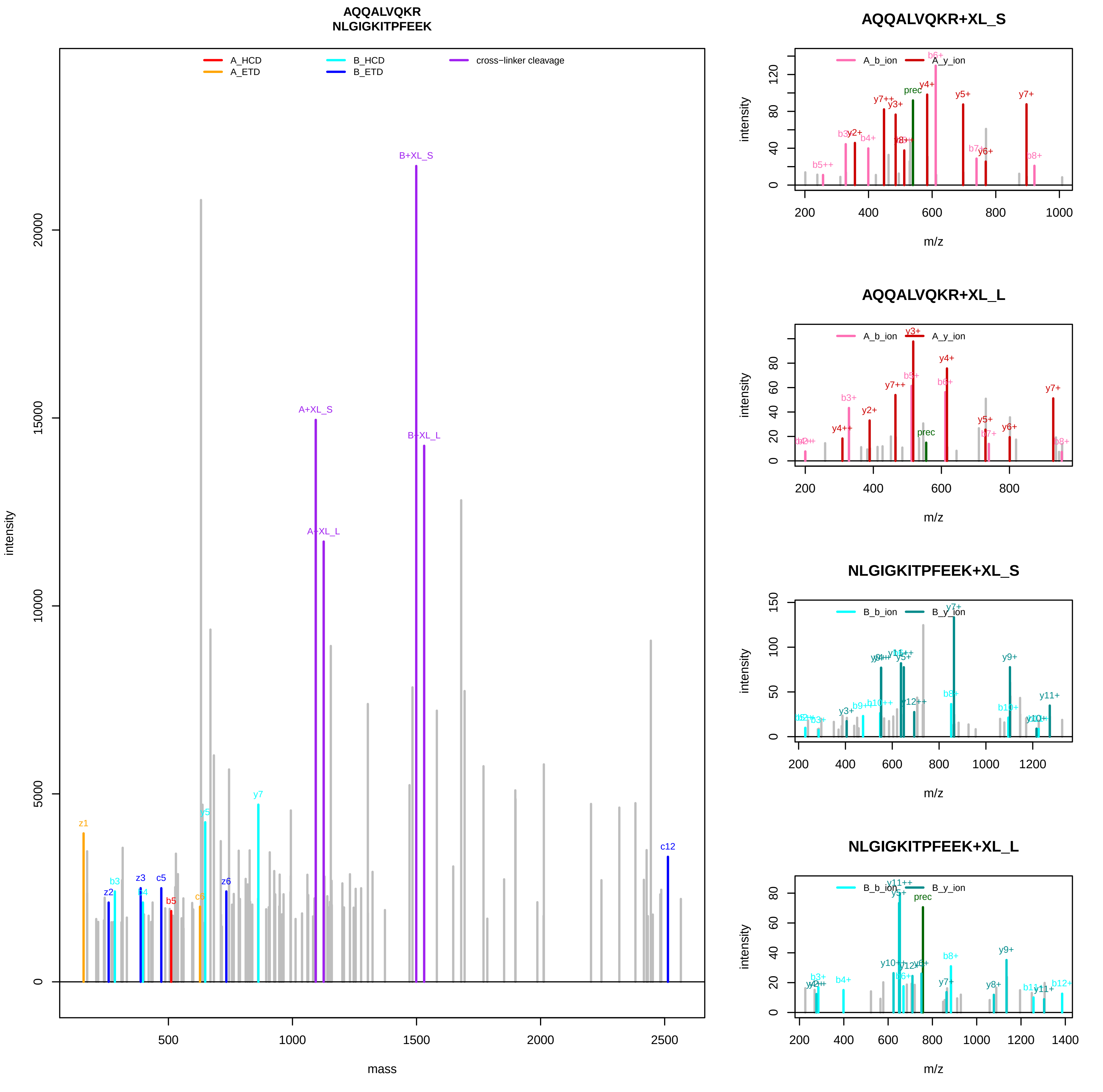
AQQALVQKR
NLGIGKITPFEEK
A_HCD
A_ETD
B_HCD
B_ETD
cross−linker cleavage
B+XL_S
A+XL_S
B+XL_L
A+XL_L
z1
z2
b3
z3
b4
c5
b5
c6
y5
z6
y7
c12
intensity
mass
0
5000
10000
15000
20000
500
1000
1500
2000
2500
AQQALVQKR+XL_S
A_b_ion
A_y_ion
prec
y7++
y3+
y4+
b6+
y5+
y7+
b3+
y2+
b4+
b5++
b7+
y6+
b8+
intensity
m/z
AQQALVQKR+XL_L
y3+
y4+
b5+
b6+
y7++
b3+
y2+
y4++
prec
y5+
b7+
y6+
y7+
b8+
NLGIGKITPFEEK+XL_S
B_b_ion
B_y_ion
y7+
y9+
y11+
y3+
b8+
b10+
y10+
NLGIGKITPFEEK+XL_L
y11++
y5+
prec
b3+
b4+
y10++
b6+
y12+
y6+
b8+
y7+
y8+
y9+
y11+
b12+
0
20
40
60
80
100
120
150
200
400
600
800
1000
1200
1400
